# Human Cytomegalovirus Clinical Strain-Specific microRNA miR-UL148D Targets the Human Chemokine RANTES during Infection

**DOI:** 10.1371/journal.ppat.1002577

**Published:** 2012-03-08

**Authors:** Youngkyun Kim, Sanghyun Lee, Sungchul Kim, Donghyun Kim, Jin-Hyun Ahn, Kwangseog Ahn

**Affiliations:** 1 National Creative Research Initiatives Center for Antigen Presentation, Department of Biological Sciences, Seoul National University, Seoul, South Korea; 2 Department of Molecular Cell Biology, Samsung Biomedical Research Institute, Sungkyunkwan University School of Medicine, Suwon, South Korea; University of Texas Health Science Center San Antonio, United States of America

## Abstract

The human cytomegalovirus (HCMV) clinical strain Toledo and the attenuated strain AD169 exhibit a striking difference in pathogenic potential and cell tropism. The virulent Toledo genome contains a 15-kb segment, which is present in all virulent strains but is absent from the AD169 genome. The pathogenic differences between the 2 strains are thought to be associated with this additional genome segment. Cytokines induced during viral infection play major roles in the regulation of the cellular interactions involving cells of the immune and inflammatory systems and consequently determine the pathogenic outcome of infection. The chemokine RANTES (Regulated on activation, normal T-cell expressed and secreted) attracts immune cells during inflammation and the immune response, indicating a role for RANTES in viral pathogenesis. Here, we show that RANTES was downregulated in human foreskin fibroblast (HFF) cells at a later stage after infection with the Toledo strain but not after infection with the AD169 strain. miR-UL148D, the only miRNA predicted from the UL/b' sequences of the Toledo genome, targeted the 3′-untranslated region of RANTES and induced degradation of RANTES mRNA during infection. While wild-type Toledo inhibited expression of RANTES in HFF cells, Toledo mutant virus in which miR-UL148D is specifically abrogated did not repress RANTES expression. Furthermore, miR-UL148D-mediated downregulation of RANTES was inhibited by treatment with a miR-UL148D-specific inhibitor designed to bind to the miR-UL148D sequence via an antisense mechanism, supporting the potential value of antisense agents as therapeutic tools directed against HCMV. Our findings identify a viral microRNA as a novel negative regulator of the chemokine RANTES and provide clues for understanding the pathogenesis of the clinical strains of HCMV.

## Author Summary

Unlike the attenuated HCMV strain AD169, the clinical isolates of HCMV, including the Toledo strain, are virulent and can cause disease in healthy adults. Toledo differs from AD169 in that Toledo contains a 15-kb DNA segment, encoding at least 19 ORFs and a single microRNA known as miR-UL148D. This 15-kb segment is believed to be a major determinant of the virulence and pathogenicity of the Toledo clinical strain. The CC–chemokine RANTES recruits immune cells during viral infection, suggesting that it may play a role in virus-related diseases. Here, we show that RANTES mRNA was degraded in human foreskin fibroblast cells during infection with Toledo but not during infection with AD169. The degradation of RANTES mRNA was mediated by miR-UL148D, the only viral microRNA predicted from the 15–kb segment of the Toledo genome. Accordingly, the levels of secreted RANTES in infected cells with ToledoΔmiR-UL148D in which miR-UL148D was deleted were higher than those in infected cells with Toledo. Our results reveal that a viral microRNA could be a novel potential therapeutic target and provide important insights into understanding the differences in pathogenic potential between clinical and attenuated strains.

## Introduction

Human cytomegalovirus (HCMV) is a member of the β-herpesvirus family and a ubiquitous human pathogen. After a primary infection, HCMV establishes lifelong latency, which seldom causes illness in an immunocompetent host [1], [2]. However, HCMV is an infectious pathogen that induces morbidity and mortality in immunocompromised individuals such as AIDS patients [3]. HCMV strains display different levels of virulence, tissue tropism, and pathogenicity depending on their degree of adaptation in fibroblasts. Injection of the low-passaged HCMV strain Toledo into healthy adults causes clinically apparent diseases [4], whereas adults inoculated with the attenuated HCMV AD169 or Towne strains do not manifest any clinical symptoms [5], [6]. These results indicate that the clinical Toledo strain is more virulent than the attenuated AD169 strain. Clinical and attenuated strains of HCMV also differ in their ability to render infected cells susceptible to the action of natural killer (NK) cells. Clinical strains confer a strong NK cell resistance, whereas high-passaged attenuated strains cause only marginal effects with respect to NK cell recognition [7], [8]. This suggests that the mechanisms employed to evade NK cell lysis may be lost during in vitro passage of the attenuated viruses. The complete genome of the laboratory-adapted strain AD169 has been sequenced [9]. An additional 19 viral genes (UL133 through UL151), which are absent from AD169, were found in low-passaged clinical isolates [10]. These genetic differences between attenuated strains and clinical strains may be implicated in HCMV-induced immunopathogenesis, as well as in strain-specific behaviors, such as tissue tropism and the ability to establish persistent or latent infections [11]–[13].

When such a virus infects its host, the host immune system is activated to remove the virus or virus-infected cells. One of the first lines of effector signals that attract circulating leukocytes to the site of viral infection is provided by chemokines [14]. Chemokines are small chemoattractant cytokines that are expressed and secreted during an inflammatory response. Chemokines attract specific immune cells during viral infection. In certain inflammatory reactions, proinflammatory cytokines, such as tumor necrosis factor alpha (TNF-α) and interferon gamma (IFN-γ), stimulate the expression of RANTES, which is chemotactic for T cells, eosinophils and basophils [15]. The initiation stage of viral infection, which includes particle binding and internalization, activates various responses in the host cells [14].

Viruses can manipulate the cellular interactions involving cells of the immune systems for their benefit by regulating cellular chemokines. Regulation of RANTES by HCMV has also been reported. Billstrom et al. showed that the abortive HCMV added to endothelial cells was capable of both entering into the host cells and uncoating. However, the virus was found to be incapable of repressing the expression of RANTES. The degradation of RANTES mRNA was also found in endothelial cells infected with the clinical isolate HCMV 4010 [16]. In contrast, Michelson et al. showed that the level of extracellular RANTES was reduced at a later stage after infection of AD169 in fibroblasts without mRNA degradation. An apparent discrepancy regarding degradation of RANTES mRNA between these two studies might be due to the genetic polymorphisms among HCMV strains. In particular, because the predominant function of miRNA is to induce degradation of target mRNA for reduced protein output [17], the differential expression of miRNAs among HCMV strains might account for this discrepancy.

miRNAs are a type of small RNA that regulates a variety of cellular processes [18], [19]. Mature miRNAs are single-stranded RNAs of 20–24 base pairs that are derived from longer transcripts processed by the enzymes Drosha and Dicer [20], [21]. Mature miRNA is incorporated into the RNA-induced silencing complex (RISC) during targeting of transcripts [22]. In the case of complete homology between miRNA and a target transcript, the target transcript is cleaved, whereas partial homology can lead to RNA cleavage or translational inhibition [23]. miRNAs exist in virtually all organisms including animals, plants and viruses. HCMV expresses multiple miRNAs during its infectious life cycle. By small RNA cloning and sequencing technique it was shown that HCMV expresses 9 miRNA precursors [24]. A bioinformatics analysis predicted that 2 additional miRNAs are conserved between HCMV and chimpanzee CMV [25]. Collectively, these 11 miRNA precursors in HCMV lead to 14 mature miRNAs, but their physiological functions are largely unknown. Interestingly, miR-UL148D miRNA exists in the 15-kb segment of clinical strains [24], [26] while it is not found in the attenuated AD169 strain. This suggests that miR-UL148D might play a role in the pathogenicity of the HCMV clinical strains.

In this study, we show that miR-UL148D directly targets RANTES, thereby inducing degradation of RANTES mRNA. Accordingly, the level of secreted RANTES was reduced by miR-UL148D during infection with the Toledo strain. A mutant Toledo virus with a deleted miR-UL148D gene did not repress RANTES expression. A PNA-based antisense oligonucleotide specific to a miR-UL148D reverted miR-UL148D-mediated downregulation of RANTES during HCMV infection. These results reveal that the clinical HCMV strain employ an additional miRNA-based mechanism to modulate the host immune system.

## Results

### An inverse relationship between RANTES mRNA and HCMV microRNA miR-UL148D levels

**Figure 1:**
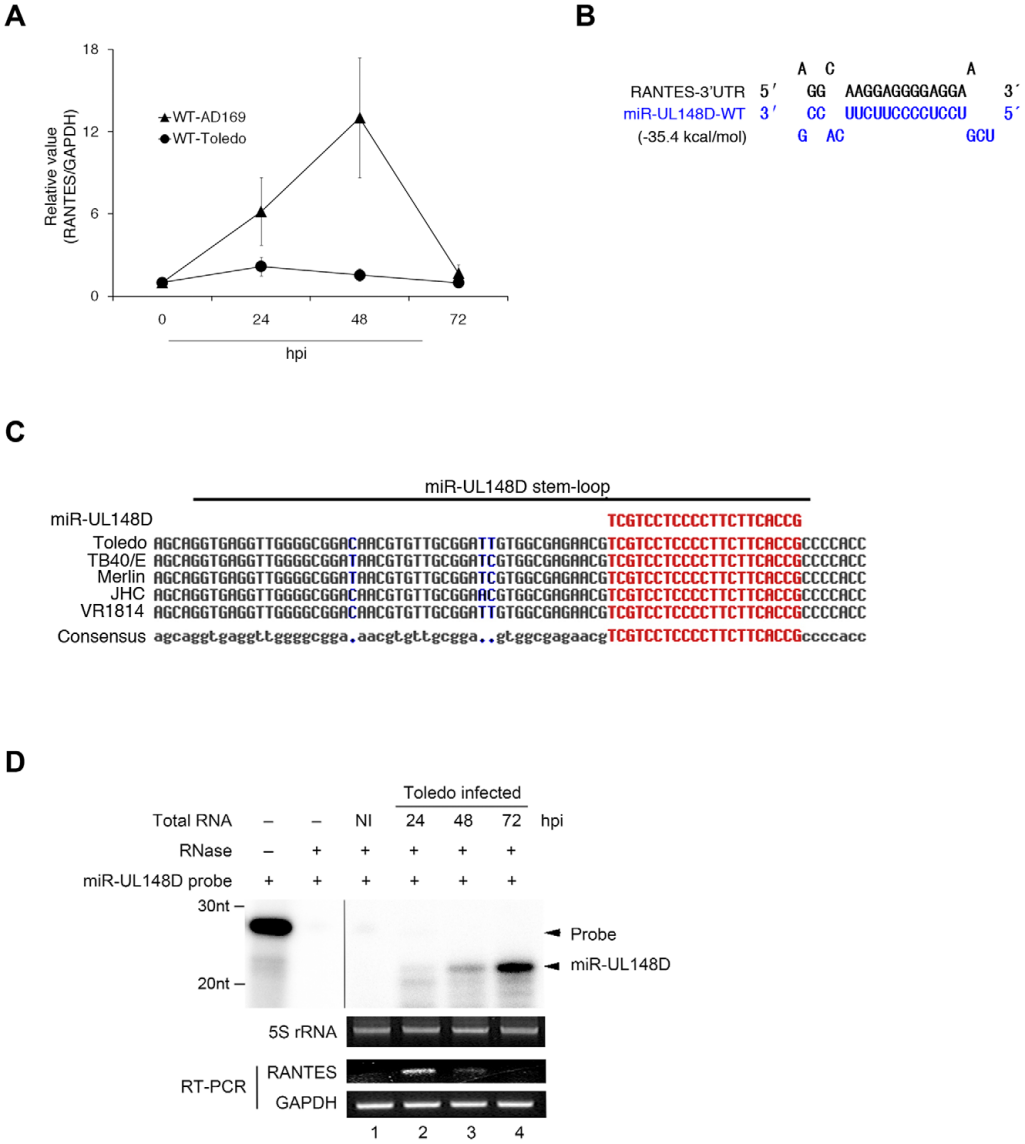
Inverse correlation between RANTES mRNA and miR-UL148D expression at a later stage during Toledo infection. (A) HFF cells were infected with WT-AD169 (triangles) or WT-Toledo (circles). RANTES mRNA at indicated time of post-infection (hpi) was quantified by quantitative RT-PCR and data were normalized to GAPDH mRNA (RANTES/GAPDH). Values represent the average ± S.D. of triplicate experiments. (B) The predicted duplex of the 3′UTR of RANTES and miR-UL148D-WT. The indicated free energy value was calculated using RNA22 (http://cbcsrv.watson.ibm.com/rna22.html). (C) Alignment of miR-UL148D stem-loop sequence of various HCMV clinical strains. We analyzed the genomic sequences of several HCMV clinical strains from GenBank (Toledo GU937742.1, TB40/E AY446866.1, Merlin AY446894.2, JHC HQ380895.1, VR1814 GU179289.1). The conserved sequence (gray) and non-conserved sequence (blue) is shown. Mature miR-UL148D sequence is highlighted in red. (D) The detection of miR-UL148D in Toledo-infected HFF cells was assessed using the RNase protection assay as described under “Materials and Methods.” 5S rRNA bands stained with ethidium bromide are presented as a loading control. Using the same RNA samples, the RANTES mRNA and GAPDH mRNA were measured by RT-PCR (lower panel). NI indicates non-infected control.

RANTES secretion is reduced by the attenuated HCMV strain AD169 in HFF cells during the replication phase [27]. Taking diverse roles of RANTES in immune and inflammatory responses into consideration and better understanding the virulence and pathogenicity of the clinical HCMV strain, we tested whether RANTES expression is also regulated by Toledo, which is the predominant clinical isolate of HCMV. HFF cells were infected with wild-type AD169 (WT-AD169) or wild-type Toledo (WT-Toledo). We employed quantitative RT-PCR to examine the RANTES mRNA levels. In the WT-AD169-infected cells, the level of RANTES mRNA increased gradually and peaked at 48 h post-infection (Figure 1A, triangles). Interestingly, WT-Toledo infection repressed the expression of RANTES mRNA throughout the infection with almost nondetectable levels at 48 h post-infection (Figure 1A, circles). These data suggest that Toledo might have at least an extra functional gene, which is lacking in AD169, that inhibits the transcription of RANTES or degrades RANTES mRNA. The primary function of miRNA is to destabilize target mRNA for reduced protein output [17]. To explore whether HCMV miRNAs are related to the regulation of the expression of RANTES, we performed bioinformatics-based target site screening. We utilized 2 previously-designed algorithms for identification of miRNA-mRNA target interaction pairs that exhibit favorable free energies (ΔG). The favorable free energies of the interaction of viral miRNAs with RANTES 3′UTR were estimated using the computational prediction algorithm, RNA22 [28]. Highly favorable free energy is provided by complementary pairing of miRNA with the 3′UTR that further stabilizes target recognition. Among the 14 miRNAs tested, only miR-UL148D [24] exhibited highly favorable free energy (ΔG = −35.4 kcal/mol). As a comparison, this interaction was predicted to be of higher stability than that observed between a pairing of a well-known let-7 miRNA with lin-28 mRNA (ΔG = −31.0 kcal/mol) (data not shown). These results were confirmed by using another algorithm, RNAhybrid [29]. This RNAhybrid algorithm yields highly favorable free energies for miR-UL148D (ΔG = −36.0 kcal/mol). On the basis of computational predictions, we concluded that miR-UL148D might target the 3′UTR of RANTES.

miR-UL148D expression has been detected by RT-PCR analysis in other clinical strains [30] and the mature miR-UL148D sequence is completely conserved among all known clinical strains (Figure 1C). We determined whether miR-UL148D is also expressed in Toledo. Total RNA was isolated from Toledo-infected HFF cells. The mature miR-UL148D was detected by RNase protection assay. RNase digestion yielded a mature miR-UL148D of ∼22 bp size that is shorter than the original miR-UL148D probe of 27-bp (Figure 1D, first panel). The expression of miR-UL148D increased throughout the infection and peaked at 72 h post-infection (Figure 1D, first panel, lanes 2–4). RT-PCR analysis of the same samples revealed that the expression level of RANTES mRNA is inversely correlated with the expression level of miR-UL148D (Figure 1D, third panel). These data suggest that Toledo-induced downregulation of RANTES may be a consequence of miR-UL148D expression.

### miR-UL148D targets the 3′UTR of RANTES

To test whether the 3′UTR of RANTES is a target of miR-UL148D, the entire RANTES 3′UTR was subcloned immediately downstream of the firefly luciferase open reading frame (ORF), and miR-UL148D was cloned into the pSuper-retro vector as a 22-nucleotide mature form. A luciferase reporter vector containing the 3′UTR of RANTES was transfected into 293T cells with varying quantities of wild-type miR-UL148D (miR-UL148D-WT) or control seed binding mutant miRNA (miR-UL148D-mut).

**Figure 2:**
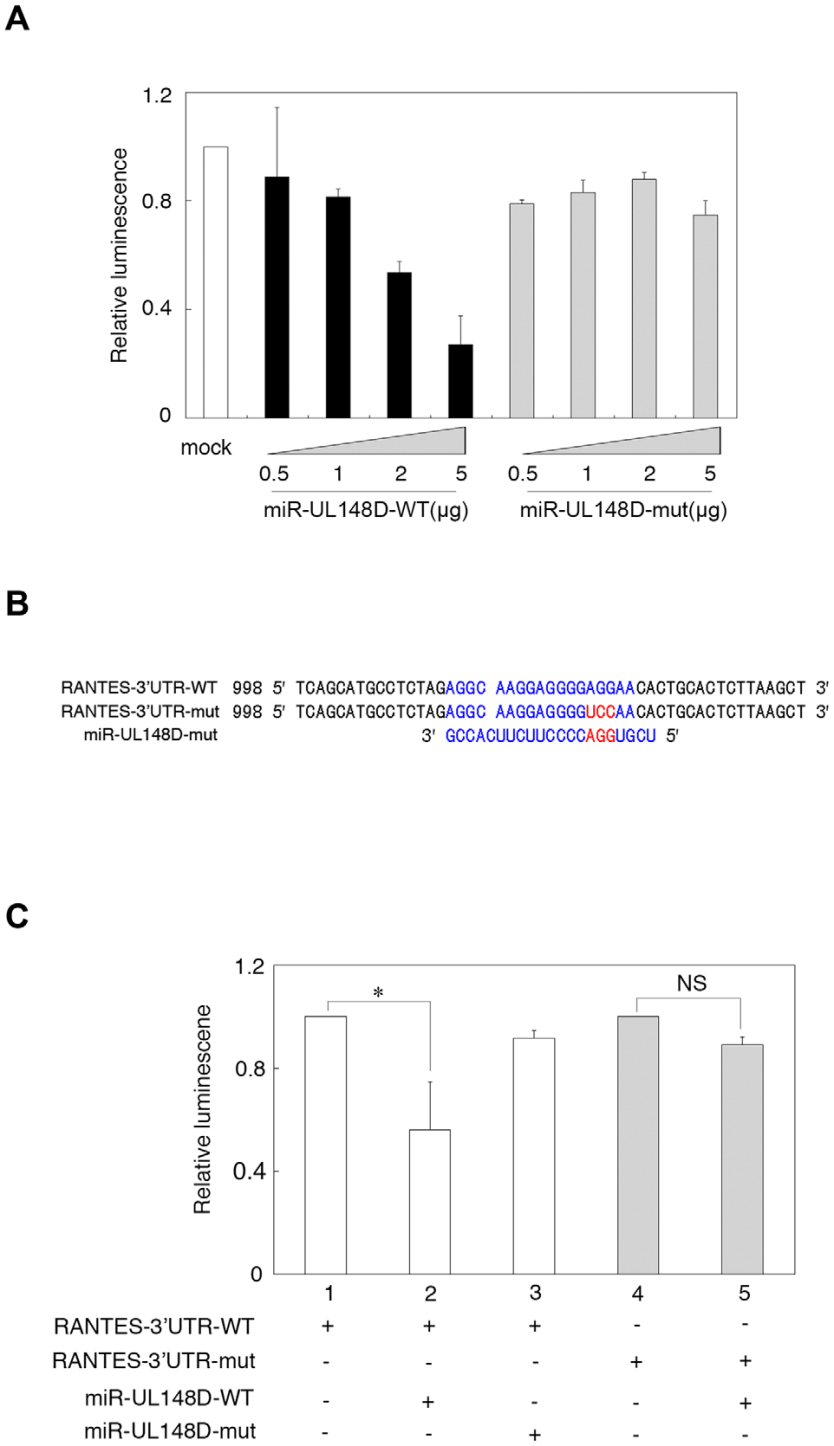
RANTES-3′UTR is a target of miR-UL148D. (A) 293T cells were co-transfected with the Renilla luciferase expression vector and firefly luciferase vector expressing normal RANTES-3′UTR (RANTES-3′UTR-WT) and either miR-UL148D, miR-UL148D-mut, or siGFP as a negative control (mock, white bar). The relative luciferase activity was calculated as a ratio of firefly to Renilla luciferase activity. (B) Mutations in the 3′-UTR of RANTES and miR-UL148D. (C) After transfection with RANTES-3′UTR-WT (white bars) or RANTES-3′UTR-mut (gray bars), cells were re-transfected with miR-UL148D-WT or miR-UL148D-mut. The cells were lysed, and firefly luciferase activity was measured and normalized to Renilla luciferase. Data represent the mean ± S.E. of four independent experiments (*statistically significant difference between cells expressing miR-control and those expressing miR-UL148D (P<0.05 by Student's t-test, NS; non-specific).

The transfected cells were lysed and appropriate substrates were added into the lysates in order to measure luciferase activity. Dose-dependent experiments demonstrated that the relative luciferase activity was significantly decreased in the presence of miR-UL148D (Figure 2A, black bars). In contrast, no reduction was observed in miR-UL148D-mut-transfected cells (Figure 2A, gray bars). Thus, the 3′UTR of RANTES has a specific binding site for miR-UL148D, indicating that it may be a target of miR-UL148D. To confirm that the 3′UTR of RANTES contains a functional target site for miR-UL148D, we generated a seed-binding mutant of the 3′UTR of RANTES (RANTES-3′UTR-mut) (Figure 2B). After co-transfection of both the RANTES 3′UTR-mut and miR-UL148D, the luciferase activity was measured. As expected, miR-UL148D-WT did not repress the luciferase activity of RANTES-3′UTR-mut-expressing cells (Figure 2C, lane 5), whereas the luciferase activity of wild type RANTES 3′UTR (RANTES-3′UTR-WT)-expressing cells was downregulated in the presence of miR-UL148D-WT (Figure 2C, lane 2). These data demonstrate that miR-UL148D targets RANTES specifically at the predicted target sequence identified in the 3′UTR.

### miR-UL148D inhibits RANTES secretion

**Figure 3:**
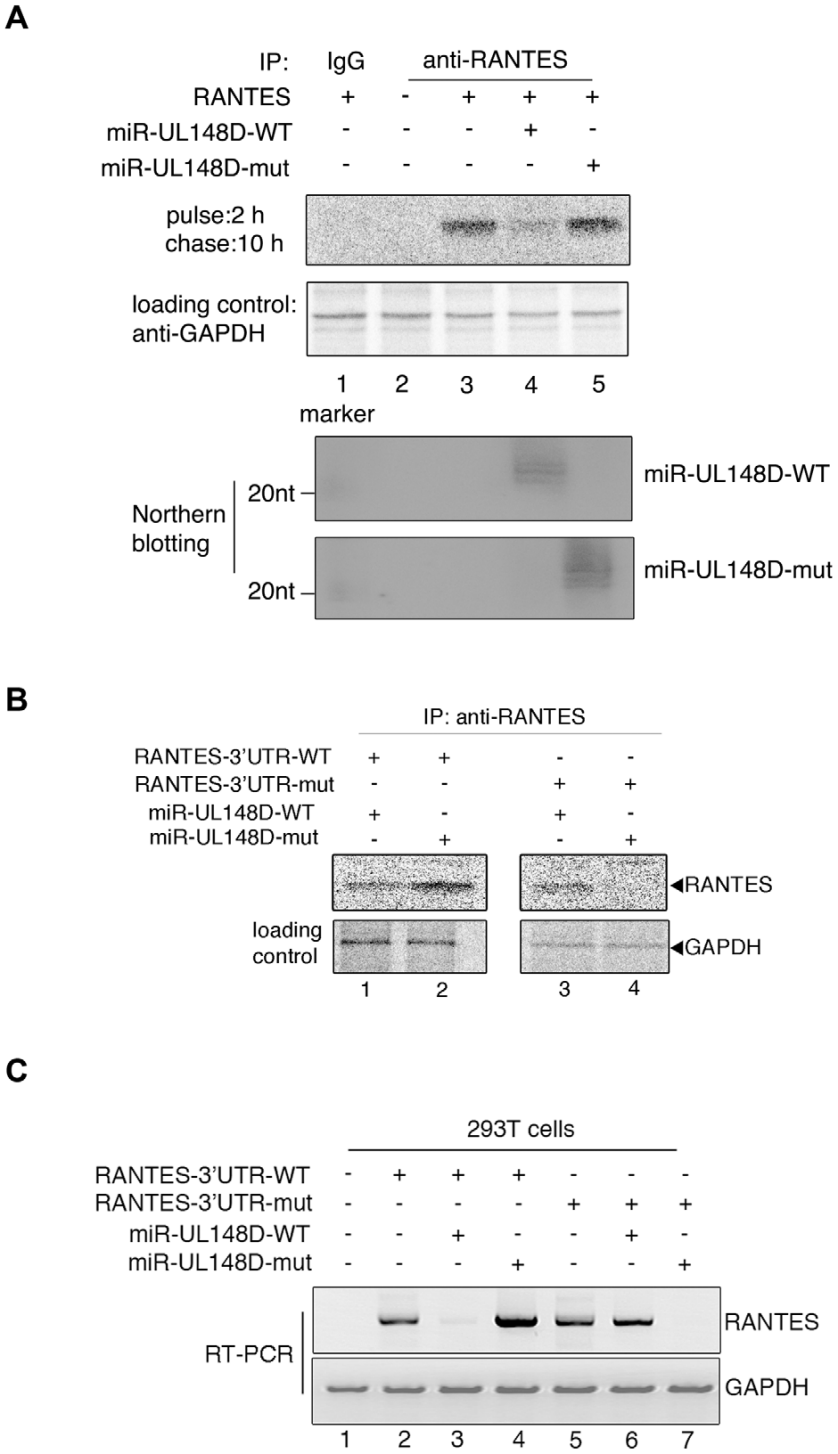
The secretion of RANTES is inhibited by miR-UL148D. (A) 293T cells were co-transfected with RANTES expressing vector including the 3′UTR and either miR-UL148D-WT or miR-UL148D-mut. The culture supernatant of the radioisotope-labeled cells was immunoprecipitated for RANTES using anti-RANTES or IgG (as an isotype control). GAPDH was immunoprecipitated by anti-GAPDH for a loading control. Northern blots were performed to confirm the expression of miRNAs. (B) 293T cells were co-transfected with both RANTES-3′UTR-WT or RANTES-3′UTR-mut and miR-UL148D-WT or miR-UL148D-mut. The culture supernatant of the isotope-labeled cells was immunoprecipitated for RANTES. GAPDH was immunoprecipitated by anti-GAPDH for a loading control. Similar results were obtained in two independent experiments. (C) mRNAs were extracted from the same samples used in Figure 3B. RANTES mRNA was measured by RT-PCR. To normalize the quantity, GAPDH mRNA was used as a loading control.

As demonstrated above, miR-UL148D represses luciferase activity in cells expressing the 3′UTR of RANTES downstream of a luciferase reporter gene (Figure 2, A and C). To ascertain whether miR-UL148D is sufficient to downregulate the level of RANTES protein, we examined the level of RANTES secreted into culture supernatants in the presence of miR-UL148D. We generated a RANTES-expressing vector which includes the 3′UTR region and tested whether the ectopic expression of miR-UL148D in 293T cells reduces the ectopic expression of RANTES. After transfection of miR-UL148D into 293T cells with the S^35^-radioisotope, secreted RANTES was immunoprecipitated with anti-RANTES antibody. Northern blot analysis validated the expression of miR-UL148D-WT and miR-UL148D-mut in transfected cells (Figure 3A, bottom panels). The amount of precipitated RANTES decreased in the presence of miR-UL148D (Figure 3A, compare lane 3 and lane 4). In contrast, miR-UL148D-mut did not affect the secretion of RANTES (Figure 3A, lane 5). These results indicate that miR-UL148D downregulates the expression of RANTES protein through its miRNA function.

Next, we determined whether the reduction of RANTES by miR-UL148D is caused by specific binding of miR-UL148D to the seed region of RANTES 3′UTR. After co-transfection of RANTES-3′UTR-WT or RANTES-3′UTR-mut with miR-UL148D-WT or miR-UL148D-mut, the labeled media of the transfected cells were immunoprecipitated with the anti-RANTES antibody. Co-transfection of both RANTES-3′UTR-WT and miR-UL148D-WT resulted in reduced levels of secreted RANTES. miR-UL148D-mut did not affect the expression of RANTES-3′UTR-WT (Figure 3B, compare lane 1 and 2). As designed, miR-UL148D-mut was able to prevent the expression of RANTES-3′UTR-mut (Figure 3B, compare lane 3 and lane 4). These results show that the 3′UTR of RANTES contains a specific target site for miR-UL148D.

To elucidate whether miR-UL148D reduces RANTES expression by translation inhibition or mRNA degradation, we determined RANTES mRNA levels in 293T cells expressing either miR-UL148D-WT or miR-UL148D-mut. RANTES-3′UTR-WT and RANTES-3′UTR-mut were transfected into 293T cells along with either miR-UL148D-WT or miR-UL148D-mut. After RNA extraction from transfected cells, RANTES mRNA levels were measured by RT-PCR. The level of RANTES-3′UTR-WT transcripts was decreased in the presence of miR-UL148D-WT but not in the presence of miR-UL148D-mut (Figure 3C, compare lane 3 and lane 4). As expected, miR-UL148D-mut targeted only RANTES-3′UTR-mut mRNA but not RANTES-3′UTR-WT for degradation (Figure 3C, compare lane 4 and lane 7). These results indicate that miR-UL148D mediates RANTES mRNA degradation but not translational repression.

### RANTES expression is repressed by miR-UL148D during authentic viral infection

We have shown that miR-UL148D encoded by an expression vector inhibits the secretion of RANTES. We determined whether the expression of RANTES is affected by miR-UL148D during viral infection. We initially utilized a mutant virus (ToledoΔUL150) in which the UL150 ORF region containing miR-UL148D was deleted. The infected cells with ToledoΔUL150 showed an increased capacity to accumulate RANTES in culture media compared with the cells infected with Toledo-WT or a revertant of ToledoΔUL150 (Figure S1).

**Figure 4:**
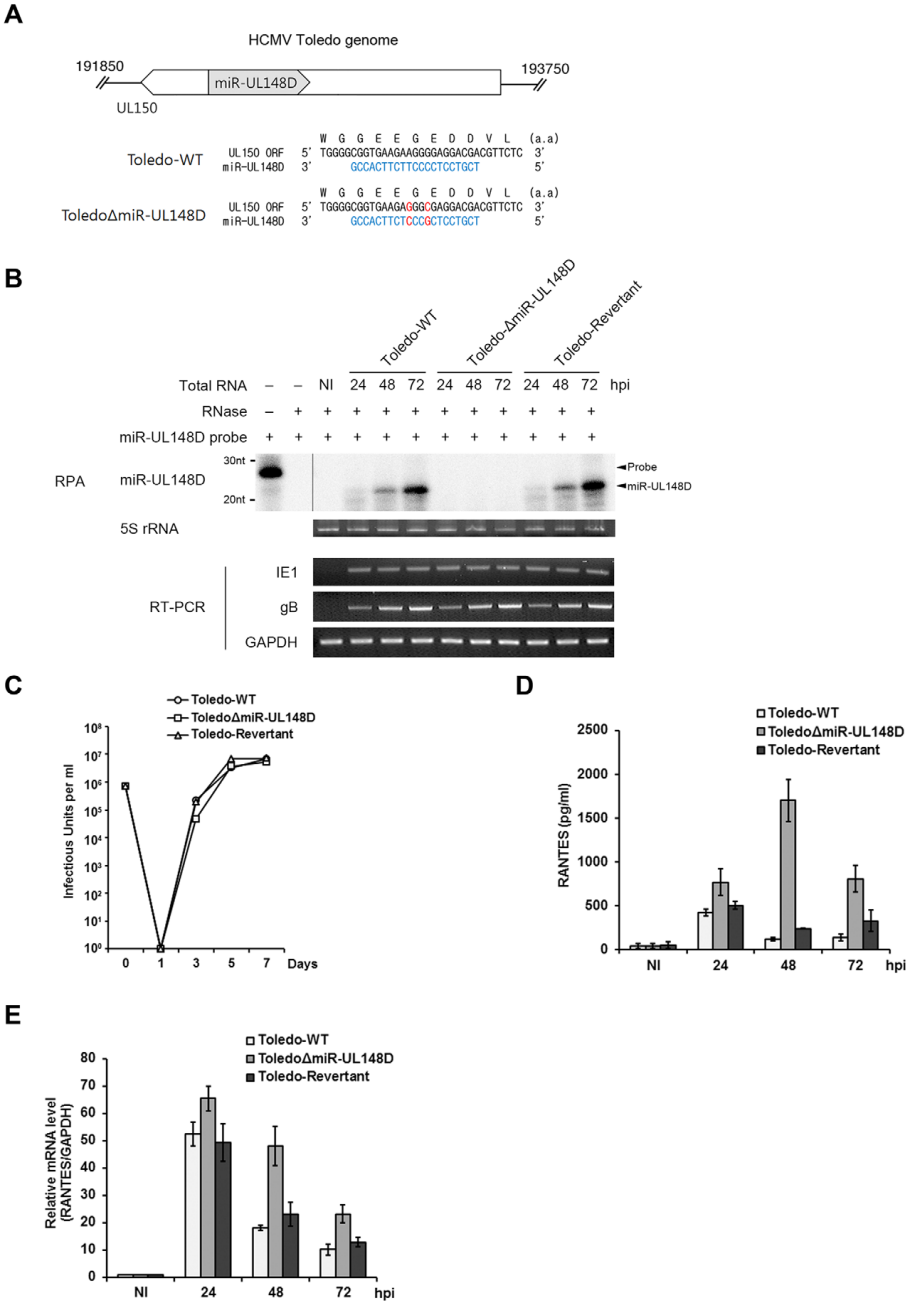
miR-UL148D inhibits RANTES expression during viral infection. (A) Genomic location of UL150 and miR-UL148D (upper panel). The predicted mature sequence of miR-UL148D (blue) and its mutated residues at wobble position are shown (red) (bottom panel). (B) HFF cells were infected with Toledo-WT, ToledoΔmiR-UL148D or Toledo-Revertant. After extracting miRNAs from the infected cells, the detection of miR-UL148D was assessed using the RNase protection assay as described under “Materials and Methods.” 5S rRNA was presented as a loading control stained by ethidium bromide. IE1 and gB gene expression was analyzed by RT-PCR. (C) Growth curves of Toledo-WT, Toledo-ΔmiR-UL148D and Toledo-Revertant. HFF cells were infected with wild-type, mutant and revertant viruses at an MOI of 2. The total number of cell-free viruses in the supernatants of infected cultures was determined by limiting dilution analyses. (D, E) After HFF cells were infected with Toledo-WT (white bars), ToledoΔmiR-UL148D (light gray bars) and Toledo-Revertant (dark gray bars), culture supernatants were harvested at the indicated post-infection time. The accumulated RANTES in supernatants was quantified by ELISA (D). RANTES mRNA level was detected by qRT-PCR (E). NI indicates non-infected control. Similar data were obtained in three independent experiments and the bars indicate mean ±S.D.

To exclude the possibility that the deletion of the UL150 ORF affected RANTES level, we generated a mutant virus (ToledoΔmiR-UL148D) in which miR-UL148D was specifically abrogated but UL150 ORF was intact and its revertant virus (Toledo-Revertant). To this aim two point mutations were engineered at the wobble positions of UL150 ORF that comprise the mature sequence of miR-UL148D (Figure 4A). RNA protection analysis confirmed that miR-UL148D was expressed in HFF cells infected with Toledo-WT or Toledo-Revertant but not with ToledoΔmiR-UL148D (Figure 4B, top panel). ToledoΔmiR-UL148D and Toledo-Revertant virus displayed phenotypes similar to the parental Toledo-WT in viral immediate-early (IE) and late (gB) gene expression and viral replication capacity (Figure 4B, bottom panels and Figure 4C). The amount of RANTES protein secreted into culture supernatants of infected HFF cells was assessed by ELISA. We found that in ToledoΔmiR-UL148D–infected cells, the amount of accumulated RANTES was significantly higher at 24–72 h post-infection than that in the Toledo-WT–infected cells (Figure 4D). qRT-PCR analysis revealed that RANTES mRNA level was also significantly higher in ToledoΔmiR-UL148D–infected cells than in Toledo-WT or Toledo-Revertant-infected cells (Figure 4E). These data demonstrate that Toledo microRNA miR-UL148D inhibits RANTES secretion by mediating degradation of RANTES mRNA during infection.

### miR-UL148D–mediated downregulation of RANTES is reversed by PNA-based antisense inhibitor during HCMV infection

**Figure 5:**
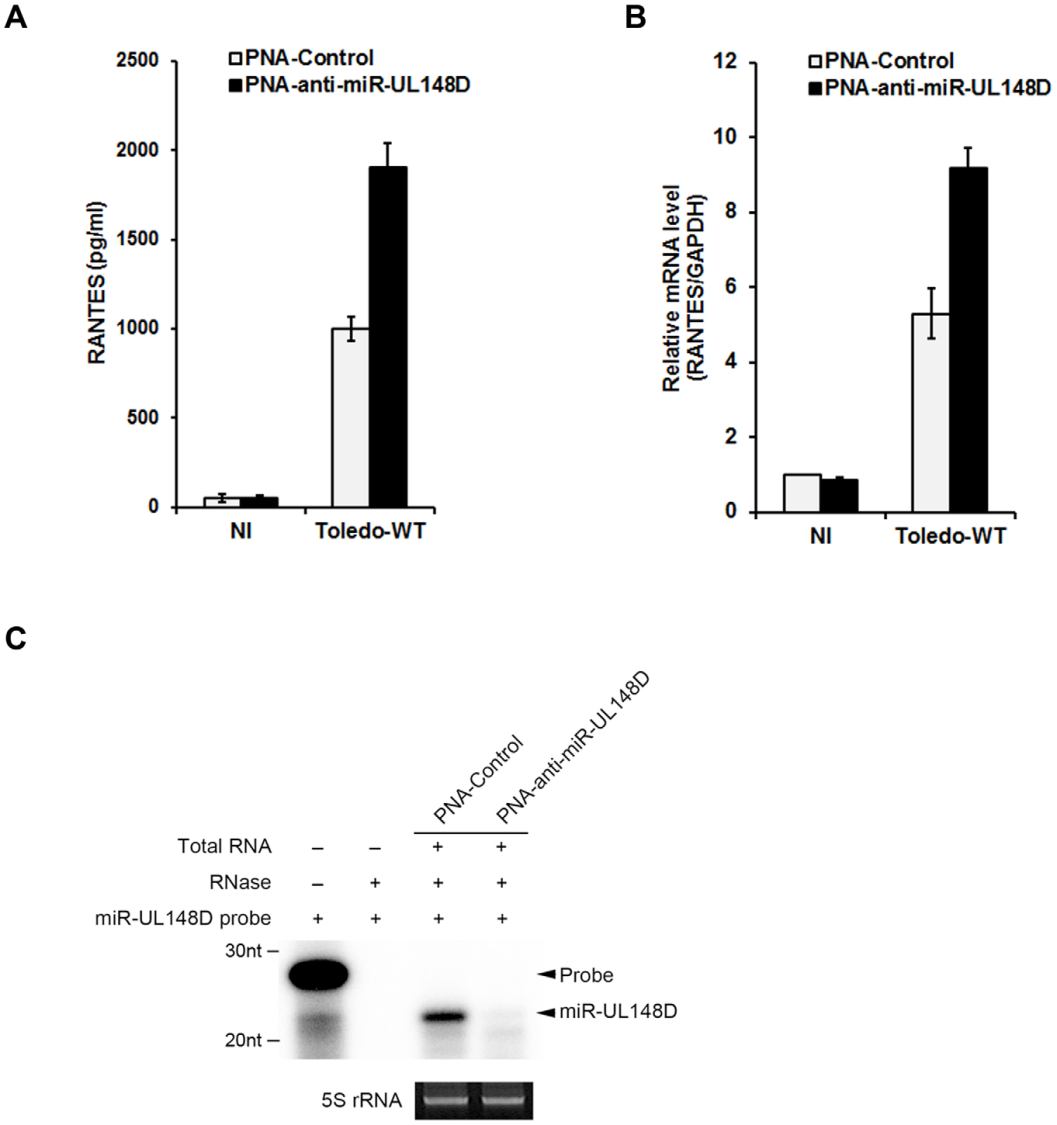
PNA-based antisense oligonucleotides specific to miR-UL148D revert Toledo-induced inhibition of RANTES production. 2 days before HCMV infection, PNA-control or PNA-anti-miR-UL148D was transfected to HFF. After 48 h of infection, culture media and total RNA were analyzed by ELISA (A) and qRT-PCR (B). Down-regulation of miR-UL148D in the presence of PNA was detected by RNase protection assay (C). NI indicates non-infected control. Similar data were obtained in three independent experiments and the bars indicate mean ±S.D.

Peptide nucleic acid (PNA), which is soluble, stable, specific to DNA or RNA and probably non-immunogenic, is known to inhibit miRNA function by an antisense mechanism through complementary binding of the PNA to the miRNA sequence [31]. To evaluate the therapeutic potential of PNA-based antisense oligonucleotides, we tested whether a miR-UL148D-specific PNA could restore RANTES expression during HCMV infection. We designed a PNA specific to miR-UL148D (PNA-anti-miR-UL148D) and a scrambled PNA (PNA-control) as a negative control. PNA-control or PNA-anti-miR-UL148D was transfected to HFF cells for 2 days before HCMV infection. At 48 h post-infection, total RNA and culture media were harvested for analysis by qRT-PCR and ELISA. In the presence of PNA-anti-miR-UL148D, secreted RANTES protein and RANTES mRNA were significantly increased (Figure 5A and B). The reduction of miR-UL148D level in HFF transfected with PNA-anti-miR-UL148D was confirmed by an RNase protection assay (Figure 5C). These results suggest that PNA-based antagonist to viral miRNA could be developed as a useful tool to counteract miRNA-based immune evasion strategies by viruses.

## Discussion

In this study, we report that infection with Toledo, a virulent clinical HCMV strain, induces degradation of RANTES mRNA in HFF cells, thereby downregulating the level of the secreted RANTES. Moreover, we show that the degradation of RANTES mRNA is mediated by HCMV-miR-UL148D, which resides in the additional DNA segment of ∼15 kb that is missing in the attenuated HCMV strains such as AD169.

Abortive HCMV added to endothelial cells is capable of both entering into the host cells and uncoating, but cannot repress the expression of RANTES [16]. In contrast, replicative HCMV infection induces cells to express significantly lower levels of RANTES [16]. These observations have led to the suggestion that HCMV encodes functional genes that can downregulate RANTES expression. In fact, HCMV appears to have developed several mechanisms for modulating RANTES at different stages of infection. The HCMV immediate-early 2 protein IE86 suppresses virus-induced RANTES expression [32]. HCMV also encodes US28 that is a receptor for RANTES. US28 binds and internalizes secreted RANTES, resulting in depletion of RANTES from the environment of HCMV-infected cells [33], [34]. In addition, HCMV encodes pUL21.5 protein that functions as a decoy receptor for RANTES. By binding to the RANTES receptor, pUL21.5 blocks the interaction of RANTES with the RANTES receptor [35]. Moreover, HCMV significantly reduces the cell-surface expression of CC chemokine receptor 1 and RANTES receptor through the internalization of receptors [36]. The multiple strategies employed by HCMV ORF gene products for RANTES regulation paradoxically underscore a critical role for RANTES in host defense against HCMV infection.

Our work identifies a viral miRNA, miR-UL148D, as a novel negative regulator for RANTES expression. To date, 14 miRNAs have been found in HCMV [24], [25], but their functions remain poorly understood. It is noteworthy that, among the 14 miRNAs, miR-UL148D is found only in clinical HCMV strains. Comparisons of nucleotide sequences revealed that the clinical Toledo genome contains a 15-kb DNA segment that is absent from the attenuated strain AD169 genome. This additional Toledo sequence encodes at least 19 ORFs and is found in all clinical strains [10]. This region is associated with strain-specific tissue tropism and immunopathogenesis [37], [38]. Interestingly, miR-UL148D exists in this genomic region of clinical strains [26].

Our findings may provide an explanation for the previous seemingly contradictory observations made by others. Michelson et al. [27] showed that the levels of both RANTES mRNA and RANTES protein are increased immediately after infection of AD169 in fibroblasts. In addition, they observed that extracellular accumulation of RANTES protein is downregulated late during HCMV infection, whereas synthesis of RANTES mRNA remains unchanged. In contrast, Billstrom and Worthen [16] found that RANTES mRNA is degraded in endothelial cells infected with the clinical isolate HCMV 4010. This result is consistent with our observations of the clinical Toledo strain (Figure 1). Our data demonstrate that the level of RANTES mRNA is downregulated in cells infected with Toledo but not with the AD169 in which an additional ∼15 kb sequence is absent. Unlike wild-type Toledo, ToledoΔmiR-UL148D virus did not cause a decrease in relative mRNA and secreted protein levels (Figure 4). Thus, our work shows that the apparent discrepancy in those studies is attributable to the genomic differences between the clinical strain and the attenuated strain, and more specifically, the presence of clinical strain-specific miR-UL148D.

High-passaged laboratory strains (AD169 and Towne) and low-passaged clinical strains (Toledo) exhibit a significant difference in their pathogenicity. For instance, Toledo grows to high titers in implanted human thymus and liver tissues in SCID-hu mice, whereas AD169 failed to replicate in human thymus and liver implants [26]. The pathogenic differences between the 2 strains are believed to be due to the existence of this additional 15-kb DNA segment. Consistent with this notion, this 15-kb genomic region encodes three envelope proteins UL128, UL130 and UL131. They form gH/gL/UL128/UL130/UL131 protein complex and the complex is required for entry in epithelial and endothelial cells [39]. Stanton et al. reported that HCMV clinical strain Merlin acquired mutations in UL128, UL130 or UL131, which inhibited virus growth specifically in fibroblast cells [40].

It is noteworthy that a few genes whose functions were described among 19 ORFs to date are related to cytokines or chemokines. The UL146 and UL147 genes encoded in this region are α-(CXC)-chemokine viral homologues, which can influence host immune responses by impairing the trafficking of peripheral blood leukocytes, particularly neutrophils [41]. UL144 encodes a homologue of the herpes simplex virus entry mediator, a member of the tumor necrosis factor (TNF)-α-like receptor superfamily [42]. However, a ligand for UL144 has not yet been identified, and the function of UL144 is still unknown [43]. Given that cytokines and chemokines are the first line of host defense and subsequently govern the downstream events of immune responses, we propose that some of the additional ORFs in the 15-kb segment could possess cytokine-related functions. Although the physiological function of UL150 is unknown, it seems that at least UL150 is not directly involved in the RANTES regulation because RANTES was detected at comparable levels in both ToledoΔmiR-UL148D and ToledoΔUL150-infected cells (Figure 4 and Figure S1). Our results demonstrate that HCMV clinical strains encode a RANTES-targeting viral miRNA. This finding adds another dimension to the reported viral immunoevasive strategies by which viral proteins have been employed to subvert the host immune responses. Considering the specificity, non-immunogenicity and relative ease of manipulation of miRNA, a better understanding of the RANTES regulation by miR-UL148D could provide the means for immunosuppressive therapy.

## Materials and Methods

### Cells and antibodies

The human embryonic kidney fibroblast cell line 293T (293T) was maintained in DMEM medium (Hyclone) containing 7.5% fetal bovine serum (HyClone Laboratories, Logan, UT), 2 mM L-glutamine, 50 U/mL penicillin, and 50 µg/mL streptomycin. Human foreskin fibroblast (HFF) cells (passage 8–10) were grown in DMEM supplemented with 10% FBS under 5% CO_2_ at 37°C. Transient transfection was performed using Lipofectamine (Invitrogen) according to the manufacturer's instructions. HEK 293T cells seeded on 60-mm culture dishes were grown to 80% confluence and transfected with proper vector using Lipofectamine 2000 (Invitrogen). After 24 h, the cells were harvested for the luciferase assay. Anti-RANTES monoclonal antibody (sc-32246) was purchased from Santa Cruz Biotechnology (Santa Cruz, CA). Anti-IE1 antibody (MAB810R) was purchased from Chemicon. Normal mouse IgG was purchased from Sigma-Aldrich.

### Virus amplification and infection

A BAC vector including the Toledo strain genome was introduced into human foreskin fibroblast cells (HFF cells) by electroporation (Bio-Rad). After 3 weeks, culture supernatants were collected and used for re-infection of fresh HFF cells. After 3 weeks, culture supernatants were collected and virus titers were measured. The virus titers were determined as infectious units after the measurement of the IE1-positive cells in the infectious center assays using HFF cells. To detect the expression of RANTES, HFF cells were infected with the Toledo strain as 2 MOI (multiple of infection).

### Dual luciferase assay

The full-length 3′-untranslated region (3′UTR) of the RANTES gene was amplified by PCR from HFF cDNA using a pair of primers (forward: GATGGAGAGTCCTTGAACCTGAAC; and reverse: TTTTTTTTTTATGGTTGCATTGAGAACTTT). Cells were co-transfected with 2.5 ng of the vector pGL3-Basic fused RANTES 3′UTR, 0.5 ng of Renilla luciferase vector, pRL-CMV (Promega) and a vector expressing miR-UL148D. Firefly activities were normalized for transfection efficiency using Renilla activity. After 16 h post-transfection, the cells were pelleted, washed in Dulbecco's phosphate-buffered saline and lysed in 1× passive lysis buffer (Promega). The firefly and Renilla luciferase activities were determined according to manufacturer's instructions (Dual-Luciferase Reporter Assay System, Promega) using a luminometer (Berthold Technologies). The relative luciferase activity was calculated from the firefly luciferase activity of RANTES 3′UTR/construct Renilla luciferase activity of co-transfected pRL-CMV)/(firefly luciferase of mock vector pGL3-Basic/Renilla luciferase activity of co-transfected pRL-CMV).

### Retrovirus production

Retroviruses encoding hairpin miRNA were constructed using the pSUPER.retro.puro vector system (OligoEngine, Seattle, WA). A double-stranded hairpin oligonucleotide designed to express the miR-UL148D (TCGTCCTCCCCTTCTTCACCG) or miR-UL148D-mut (TCGTGGACCCCTTCTTCACCG) was cloned into the BglII/HindIII sites of pSUPER.retro.puro. A control miRNA was constructed, which contains a 19-bp target sequence to target the GFP molecule. Correct insertion of the oligonucleotides was confirmed by DNA sequencing. pSuper-retro viruses were produced in Phoenix-Ampho packaging cells.

### Northern blot and RT-PCR

Extracted total RNA from HFF or 293T was resolved on 15% acrylamide, 8 M urea gel, transferred onto nylon membranes, and UV cross-linked. [γ-^32^P]ATP labeled antisense RNA as a probe for each miRNA was purchased from Cosmo Genetech (Seoul, Korea). Hybridization was performed in the hybridization solution overnight at room temperature and the FUJI BAS film was exposed for 1 day. 5S rRNA bands stained with ethidium bromide are presented as a loading control for normalization.

DNase-treated total RNA was reverse-transcribed with oligo dTs. The RT reaction was carried out at room temperature for 10 min. The reaction mixtures were then heated in a thermal cycler at 42°C for 15 min and 99°C for 5 min and then cooled at 5°C for 5 min. After adding specific primers and PCR reagents, the mixture was denatured at 95°C for 4 min. The mixture was then denatured at 95°C for 1 min, annealed at 55°C for 1 min, and extended at 72°C for 1 min. After 25 cycles, the PCR mixture was incubated at 72°C for 7 min. To confirm the identity of the amplified cDNAs, each insert product was directly sequenced using a specific primer sequence or asymmetrically cut with restriction enzymes.

The primers for RT-PCR were as follows: HCMV IE1 (forward, GTCAGGTCCACCACTGACAC; reverse, TCATATTAAAGGCGCCAGTG), HCMV gB (forward, AACGCGGCTGTAAGAACTGT; reverse, ACGAGGGCATCATGGTAGTC), GAPDH (forward, ATCATC CCTGCCTCTACTGG; reverse, GTCAGGTCCACCACTGACAC).

### RNase protection assay

Total RNA was isolated from Toledo-infected HFF cells at various post-infection times. The mirVana miRNA probe construction kit (Ambion) was used to synthesize the ^32^P-labeled miR-UL148D antisense probe (CGGUGAAGAAGGGGAGGACGACCAGAG). This probe was designed so that an obvious size difference is detectable between the full-length undigested probe and the protected fragment after RNase digestion. Probe hybridization and RNase protection was then carried out using the mirVana miRNA detection kit (Ambion) according to the manufacturer's instructions. Briefly, the total RNA was incubated at 42°C for hybridization to the miR-UL148D-specific probe. After hybridization, unhybridized RNA and excess RNA probe were removed by RNase digestion. The double-stranded product was resolved in a 12% polyacrylamide, 8 M urea denaturing gel and visualized using autoradiography.

### Quantitative real-time PCR

RNA isolation from HCMV-infected cells was performed according to the manufacturer's instructions (Invitrogen). Viral RNA was quantified by qRT-PCR using the iScript cDNA synthesis kit (Bio-Rad) for 2 h at 42°C. cDNA was amplified with human RANTES-specific primers (forward, CTCATTTGCTACTGC CCTCTGCGCTCCTGC; reverse, GCTCATCTCCAAAGAGTTGATGTACTC), HCMV IE1-specific primers (forward, GTCAGGTCCACCACTGACAC; reverse, TCATATTAAAGGCGCCAGTG), and GAPDH-specific primers (forward, ATCATC CCTGCCTCTACTGG; reverse, GTCAGGTCCACCACTGACAC). Quantification of HCMV RNA was normalized with GAPDH RNA as an internal control. Reactions were performed by the Lightcycler PCR (Roche) using the following program: 50°C for 30 min, 95°C for 15 min and 45 cycles as follows: 95°C for 10 s, 53°C for 10 s, and 72°C for 25 s.

### Computational prediction

The online target prediction algorithm RNA22 (http://cbcsrv.watson.ibm.com/rna22.html) and RNAhybrid (http://bibiserv.techfak.uni-bielefeld.de/rnahybrid/submission.html) were used to predict potential target sites. HCMV 14 miRNAs were used as target miRNAs [24], [44].

### Viral mutagenesis

To generate the deletion mutant virus of the miR-UL148D (ToledoΔmiR-UL148D), UL150 region encoding miR-UL148D was deleted in the Toledo bacterial artificial chromosome (BAC) using rpsL-neo cassettes. Briefly, rpsL-neo cassettes were PCR amplified by using primers containing homology arms consisting of 50 nucleotides upstream and downstream of the target gene plus 24 nucleotides homologous to the rpsL-neo cassette. The amplified DNA fragments were introduced into E. coli DH10b cells containing wild-type Toledo-BAC for recombination by electroporation using Gene Pulser II (Bio-Rad). The intermediate Toledo-BAC construct containing the rpsL-neo cassette was selected on Luria broth (LB) plates containing kanamycin. UL150 region in Toledo-BAC was destroyed by insertion of rpsL-neo cassette. We also generated Toledo-Revertant BAC by the same method. Deletion of miR-UL148D was confirmed by RNase protection assay using specific probe.

### Transfection of miRNA inhibitor PNA

PNA miRNA inhibitors were purchased from Panagene Inc. The miR-UL148D-specific inhibitor sequence is 5′-RRRQRRKKR-OO-GTGAAGAAGGGGAGGACG-3′ and the scrambled control PNA sequence is 5′-RRRQRRKKR-OO-TAGAGCTCCCTTCAATCCAAA-3′. One day before transfection, HFF cells were seeded onto a 24-well plate in appropriate complete growth medium without antibiotics. PNAs transfection was performed using Dharmafect 1 reagent (Dharmacon). PNA miRNA inhibitor was added into the culture medium at a final concentration of 500∼2000 nM. Cells were incubated at 37°C for 48 h prior to viral infection. A miRNA isolation kit (Invitrogen) was used to isolate miRNA from total RNA.

### Enzyme-linked immunosorbent assays (ELISA)

ELISA was used to measure the expression level of secreted RANTES. A RANTES ELISA kit was purchased from Thermo Fisher Scientific Inc. (Rockford, IL). Culture media of transfected or infected cells were collected and incubated with RANTES antibody on the plate. Each well was washed with the washing buffer provided in the ELISA kit. After incubation of HRP-conjugated antibody, TMB substrate was added into each well. Absorbance was measured using a microplate reader, iMARK (Bio-Rad) at a wavelength of 450 nm.

### Immunoprecipitation

Cells were labeled with [^35^S]methionine and [^35^S]cysteine and lysed in 1% NP-40 in PBS supplemented with protease inhibitors. After preclearing, samples were incubated with the appropriate antibodies for 2 h at 4°C before Protein G-Sepharose beads were added. Beads were washed four times with 0.1% NP-40 in PBS and bound proteins were eluted by boiling in SDS sample buffer. Proteins were separated by SDS-PAGE.

## Supporting Information

Figure S1 **Generation of mutant virus lacking the UL150 ORF region that contains miR-UL148D.** (A) A schematic diagram of the ToledoΔUL150 and its revertant BAC construct. (B) Deletion of miR-UL148D was confirmed by RNase protection assay using specific probe (upper panel). IE gene expression was analyzed by immunobloting (bottom panel). (C) The amount of secreted RANTES in culture media was determined by ELISA.
(TIF)
