# Supplementary figures and images for: Human Cytomegalovirus Clinical Strain-Specific microRNA miR-UL148D Targets the Human Chemokine RANTES during Infection
Source: PLoS Pathog. 2012 Mar 8;8(3):e1002577. doi: 10.1371/journal.ppat.1002577 (PMC3297591; doi:10.1371/journal.ppat.1002577)

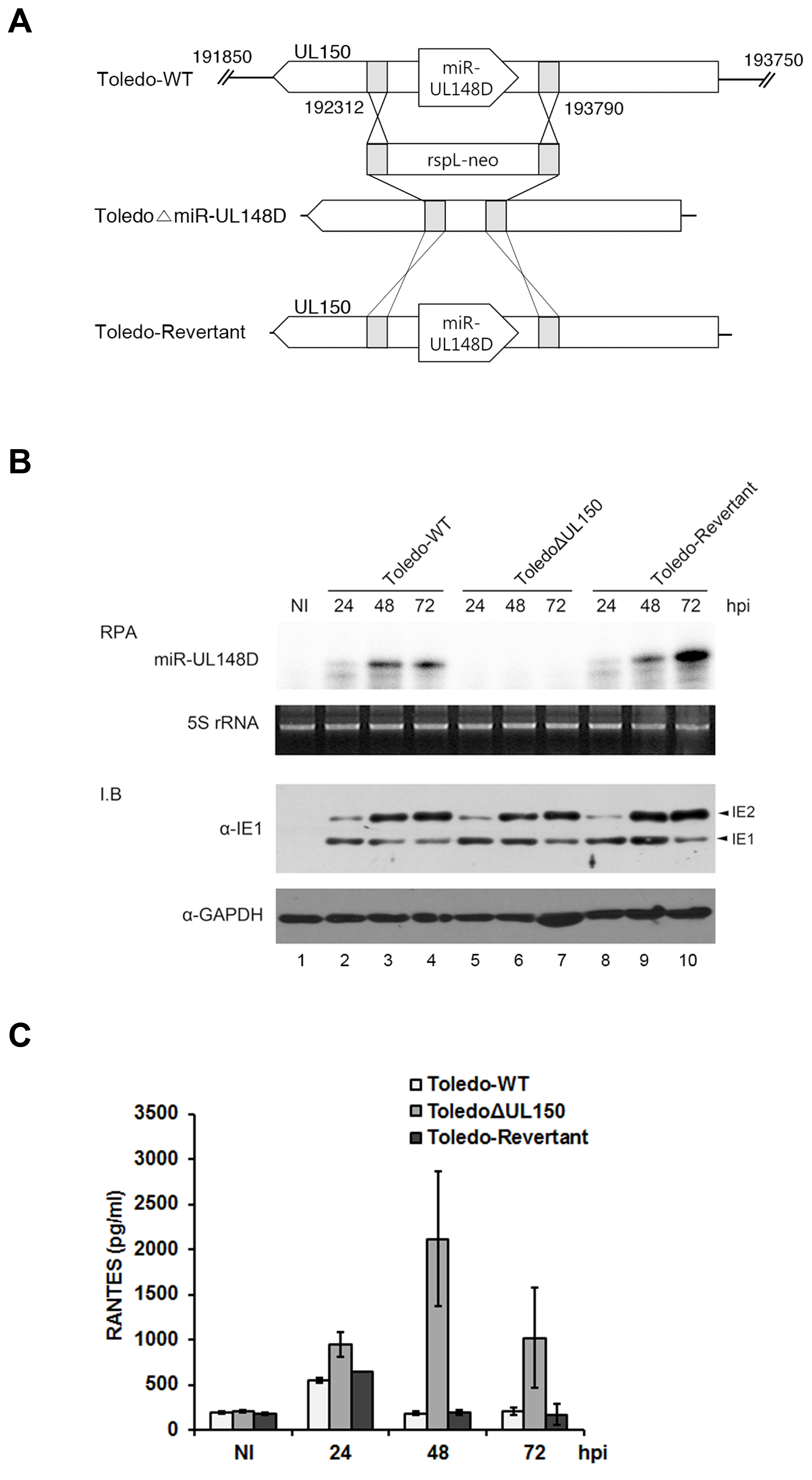

Supplement: Figure S1 — Generation of mutant virus lacking the UL150 ORF region that contains miR-UL148D. (A) A schematic diagram of the ToledoΔUL150 and its revertant BAC construct. (B) Deletion of miR-UL148D was confirmed by RNase protection assay using specific probe (upper panel). IE gene expression was analyzed by immunobloting (bottom panel). (C) The amount of secreted RANTES in culture media was determined by ELISA. (TIF) [file ppat.1002577.s001.tif]
